# Impact of homocysteine levels on mortality risk in patients with chronic limb-threatening ischemia undergoing revascularization

**DOI:** 10.1007/s00380-021-01877-0

**Published:** 2021-06-15

**Authors:** Mitsuyoshi Takahara, Osamu Iida, Yoshimitsu Soga, Akio Kodama, Hiroto Terashi, Nobuyoshi Azuma

**Affiliations:** 1 Department of Metabolic Medicine, Osaka University Graduate School of Medicine, 2-2 Yamadaoka, Suita, 565-0871 Japan; 2 Department of Diabetes Care Medicine, Osaka University Graduate School of Medicine, 2-2 Yamadaoka, Suita, 565-0871 Japan; 3 Cardiovascular Center, Kansai Rosai Hospital, 3-1-69 Inabaso, Amagasaki, Hyogo 660-8511 Japan; 4 Department of Cardiology, Kokura Memorial Hospital, 3-2-1 Asano, Kokurakita-ku, Kitakyushu, 802-0001 Japan; 5 Division of Vascular Surgery, Department of Surgery, Nagoya University Graduate School of Medicine, 65 Tsurumai-cho, Showa-ku, Nagoya, 466-8550 Japan; 6 Department of Plastic Surgery, Kobe University Graduate School of Medicine, 7-5-2, Kusunoki-cho, Chuo-ku, Kobe, 650-0017 Japan; 7 Department of Vascular Surgery, Asahikawa Medical University, 2-1 Midorigaoka-higashi, Asahikawa, 078-8510 Japan

**Keywords:** Chronic limb-threatening ischemia, Homocysteine levels, Renal function, Cystatin C, Mortality risk

## Abstract

The current study aimed to reveal the clinical impact of plasma homocysteine levels in chronic limb-threatening ischemia (CLTI) patients undergoing revascularization. This was a sub-analysis of a prospective multicenter registry of CLTI patients, named the Surgical reconstruction versus Peripheral INtervention in pAtients with critical limb isCHemia (SPINACH) study. The current analysis included 192 non-dialysis-dependent CLTI patients who underwent revascularization for CLTI, and whose plasma homocysteine levels at baseline were available. The association of clinical characteristics with homocysteine levels was evaluated with the linear regression model. The association of homocysteine levels with the mortality risk was investigated using the Cox proportional hazards regression model. Cystatin C-based estimated glomerular filtration rate (eGFR) was independently associated with log-transformed homocysteine levels; the adjusted standardized regression coefficient (95% confidence interval) was − 0.432 (− 0.657 to − 0.253; *P* < 0.001). Homocysteine levels were significantly associated with the mortality risk in the univariate model (*P* = 0.017); the unadjusted hazard ratio was 1.71 (1.13–2.50) per twofold increase. The association was significantly attenuated when adjusted for cystatin C-based eGFR (*P* < 0.001); the hazard ratio adjusted for cystatin C-based eGFR was 1.28 (0.80–1.90; *P* = 0.29). An apparent association of homocysteine levels with an increased risk of mortality could be explained by renal dysfunction. Future studies will be needed to validate the current findings.

## Introduction

Chronic limb-threatening ischemia (CLTI) is the most advanced form of peripheral arterial disease, and patients with CLTI are at high risk of mortality, even after undergoing a timely revascularization [1]. Hyperhomocysteinemia is a well-known risk factor for atherosclerosis, especially for peripheral artery disease [2–4]. While the benefit of its correction by folate supplementation appear to be negligible [2–4], hyperhomocysteinemia per se has been regarded as a classic, useful biomarker for the risk of peripheral artery disease [2–4], and for clinical outcomes in a population with peripheral artery disease [5–7]. However, little was known about its impact on the mortality risk in CLTI patients undergoing revascularization.

The aim of the current study was to reveal the clinical impact of plasma homocysteine levels in CLTI patients undergoing revascularization.

## Materials and methods

We used a clinical database obtained from the Surgical reconstruction versus Peripheral INtervention in pAtients with critical limb isCHemia (SPINACH) study, a prospective, multicenter, observational study that registered patients with CLTI due to atherosclerotic arterial disease at 23 centers (12 vascular surgery departments and 11 interventional cardiology departments) in Japan [1, 8]. CLTI patients were registered at the referral to the participating centers, between January 2012 and March 2013, and were scheduled to be followed-up for 3 years. The details of the SPINACH study are described elsewhere [1, 8]. The study was performed in accordance with the Declaration of Helsinki and was approved by the ethics committee at the principal research institution, Asahikawa University Hospital (No. 1023) and all the other centers registering patients. Written informed consent was obtained from every participant. The current analysis included 192 non-dialysis-dependent CLTI patients who underwent revascularization for either ischemic wound with the Wound, Ischemia, and foot Infection (WIfI) classification system [9] Ischemia (I) grade 2/3 or ischemic rest pain with the WIfI I-3, and whose plasma homocysteine levels at baseline were available. Surgical reconstruction and endovascular therapy alone were selected in 69 and 122 patients, respectively. Skin perfusion pressures of 31–40 mmHg and ≤ 30 mmHg were treated as WIfI I-2 and 3, respectively [1]. The estimated glomerular filtration rate (eGFR) was calculated from the creatinine equation [10] and from the cystatin C equation [11].

### Statistical analysis

Data are given as means and standard deviations or medians and interquartile ranges for continuous variables or as frequencies and percentages for discrete variables, respectively, if not otherwise mentioned. A *P* value of less than 0.05 was considered statistically significant, and 95% confidence intervals are reported where appropriate. The association of clinical characteristics with homocysteine levels were evaluated with the linear regression model in which log-transformed homocysteine levels were treated as the dependent variable. The association with log-transformed homocysteine levels with the risk of all-cause mortality was investigated using the Cox proportional hazards regression model. We obtained the unadjusted hazard ratio from the univariate model, and the hazard ratio adjusted for each clinical characteristic from the bivariate model in which homocysteine levels and a covariate of interest were entered together. We additionally obtained the hazard ratio of log-transformed homocysteine levels for all-cause mortality with simultaneous adjustment for age, diabetes, smoking, and creatinine- and cystatin C-based eGFRs, using the multivariate Cox proportional hazards regression model. The association of homocysteine levels with the risk of major amputation and that of major adverse limb events (defined as a composite of major amputation and any reintervention) was analyzed using Fine and Gray’s proportional hazards regression model for the sub-distribution of competing risks. Missing data were addressed using the multiple imputation by chained equations method. P values and 95% confidence intervals were obtained from 2000-time bootstrap resampling. All statistical analyses were performed using R version 3.6.0 (R Development Core Team, Vienna, Austria).

## Results

Clinical characteristics of the study population are summarized in Table 1. Mean age was 75 ± 10 years. Ischemic rest pain accounted for 15.2% and ischemic tissue loss accounted for the remaining 84.8%. Median plasma homocysteine levels (interquartile range) were 12.8 (10.0–18.1) nmol/mL. Sixty-nine patients (36.1%) underwent surgical reconstruction, and the remaining 122 (63.9%) underwent endovascular therapy. As shown in Table 2, creatinine- and cystatin C-based eGFRs had a crude inverse association with log-transformed homocysteine levels; the unadjusted standardized regression coefficient (95% confidence interval) was − 0.372 (− 0.513 to − 0.235) and − 0.467 (− 0.592 to − 0.336), respectively (both *P* < 0.001). In the multivariate regression model, cystatin C-based eGFR, but not creatinine-based eGFR or the other baseline characteristics, had an independent association with log-transformed homocysteine levels (Table 2).

**Table 1:** Clinical characteristics of the study population

n	191
Age (years)	75 ± 10
Male sex	121 (63.4%)
Impaired mobility	
None (self-ambulatory)	116 (60.7%)
Requiring equipment	66 (34.6%)
Requiring personal aid	9 (4.7%)
Body mass index (kg/m^2^)	21.9 ± 3.4
Smoking history	
Never	77 (40.3%)
Past	78 (40.8%)
Current	36 (18.8%)
Diabetes mellitus	128 (67.0%)
Left ventricular ejection fraction (%)	64 ± 11
(Missing data)	7 (3.7%)
Creatinine-based eGFR (mL/min/1.73 m^2^)	60 ± 28
Cystatin C-based eGFR (mL/min/1.73 m^2^)	55 ± 24
(Missing data)	13 (6.8%)
Hemoglobin levels (g/dL)	11.6 ± 2.1
Albumin levels (g/dL)	3.5 ± 0.6
(Missing data)	1 (0.5%)
Cholinesterase (U/L)	239 ± 75
(Missing data)	4 (2.1%)
Homocysteine levels (nmol/mL)	12.8 (10.0–18.1)
Medication use	
Statin use	59 (30.9%)
Renin-angiotensin system inhibitor use	102 (53.4%)
Beta blocker use	36 (18.8%)
Aspirin use	118 (61.8%)
Thienopyridine use	57 (29.8%)
Cilostazol use	63 (33.0%)
Insulin use	40 (20.9%)
Oral antidiabetic medication use	59 (30.9%)
History of intermittent claudication	116 (61.1%)
(Missing data)	1 (0.5%)
WIfI Wound grade	
W-0	29 (15.2%)
W-1	53 (27.7%)
W-2	80 (41.9%)
W-3	29 (15.2%)
WIfI Ischemia grade	
I-2	28 (14.7%)
I-3	163 (85.3%)
WIfI Foot infection grade	
fI-0	106 (55.5%)
fI-1	48 (25.1%)
fI-2	36 (18.8%)
fI-3	1 (0.5%)
Bilateral CLTI	28 (14.7%)
Revascularization strategy	
Surgical reconstruction	69 (36.1%)
Endovascular therapy	122 (63.9%)

Data are mean ± standard deviation, median (interquartile range) or frequency (percentage)

**Table 2:** Association of clinical characteristics with homocysteine levels

	Unadjusted standardized regression coefficients	Adjusted standardized regression coefficients
Age	0.037 [− 0.131 to 0.203] (*P* = 0.67)	− 0.099 [− 0.270 to 0.072] (*P* = 0.28)
Male sex	− 0.033 [− 0.183 to 0.110] (*P* = 0.66)	0.026 [− 0.154 to 0.178] (*P* = 0.78)
Body mass index	− 0.013 [− 0.160 to 0.137] (*P* = 0.89)	0.008 [− 0.118 to 0.148] (*P* = 0.87)
Impaired mobility	0.066 [− 0.112 to 0.239] (*P* = 0.48)	0.071 [− 0.098 to 0.235] (*P* = 0.46)
Smoking	0.025 [− 0.115 to 0.167] (*P* = 0.71)	0.086 [− 0.068 to 0.261] (*P* = 0.26)
Diabetes mellitus	0.021 [− 0.126 to 0.166] (*P* = 0.77)	− 0.015 [− 0.155 to 0.123] (*P* = 0.83)
Left ventricular ejection fraction	− 0.034 [− 0.146 to 0.086] (*P* = 0.59)	0.069 [− 0.048 to 0.190] (*P* = 0.26)
Creatinine-based eGFR	− 0.372 [− 0.513 to − 0.235] (*P* < 0.001)	− 0.092 [− 0.298 to 0.152] (*P* = 0.46)
Cystatin C-based eGFR	− 0.467 [− 0.592 to − 0.336] (*P* < 0.001)	− 0.432 [− 0.657 to − 0.253] (*P* < 0.001)
Hemoglobin levels	− 0.094 [− 0.211 to 0.021] (*P* = 0.11)	0.009 [− 0.123 to 0.155] (*P* = 0.84)
Albumin levels	− 0.072 [− 0.193 to 0.056] (*P* = 0.28)	0.036 [− 0.133 to 0.212] (*P* = 0.65)
Cholinesterase levels	− 0.073 [− 0.219 to 0.072] (*P* = 0.34)	− 0.032 [− 0.213 to 0.143] (*P* = 0.70)
History of claudication	− 0.138 [− 0.281 to 0.007] (*P* = 0.061)	− 0.077 [− 0.208 to 0.067] (*P* = 0.28)
WIfI-W grade	0.060 [− 0.099 to 0.223] (*P* = 0.47)	0.065 [− 0.126 to 0.246] (*P* = 0.53)
WIfI-I grade	− 0.095 [− 0.250 to 0.059] (*P* = 0.22)	− 0.066 [− 0.205 to 0.087] (*P* = 0.43)
WIfI-fI grade	− 0.103 [− 0.247 to 0.045] (*P* = 0.17)	− 0.136 [− 0.279 to 0.021] (*P* = 0.094)
Bilateral CLTI	0.129 [− 0.056 to 0.310] (*P* = 0.16)	0.122 [− 0.037 to 0.274] (*P* = 0.13)
Endovascular therapy vs surgical reconstruction	0.102 [− 0.026 to 0.231] (*P* = 0.10)	0.061 [− 0.076 to 0.189] (*P* = 0.37)

Data are presented as standardized regression coefficients for log-transformed homocysteine levels [95% confidence intervals] (*P* values). Adjusted standardized regression coefficients were obtained from the multivariate linear model in which all the variables listed in the table were entered as the explanatory variables

**Fig. 1:**
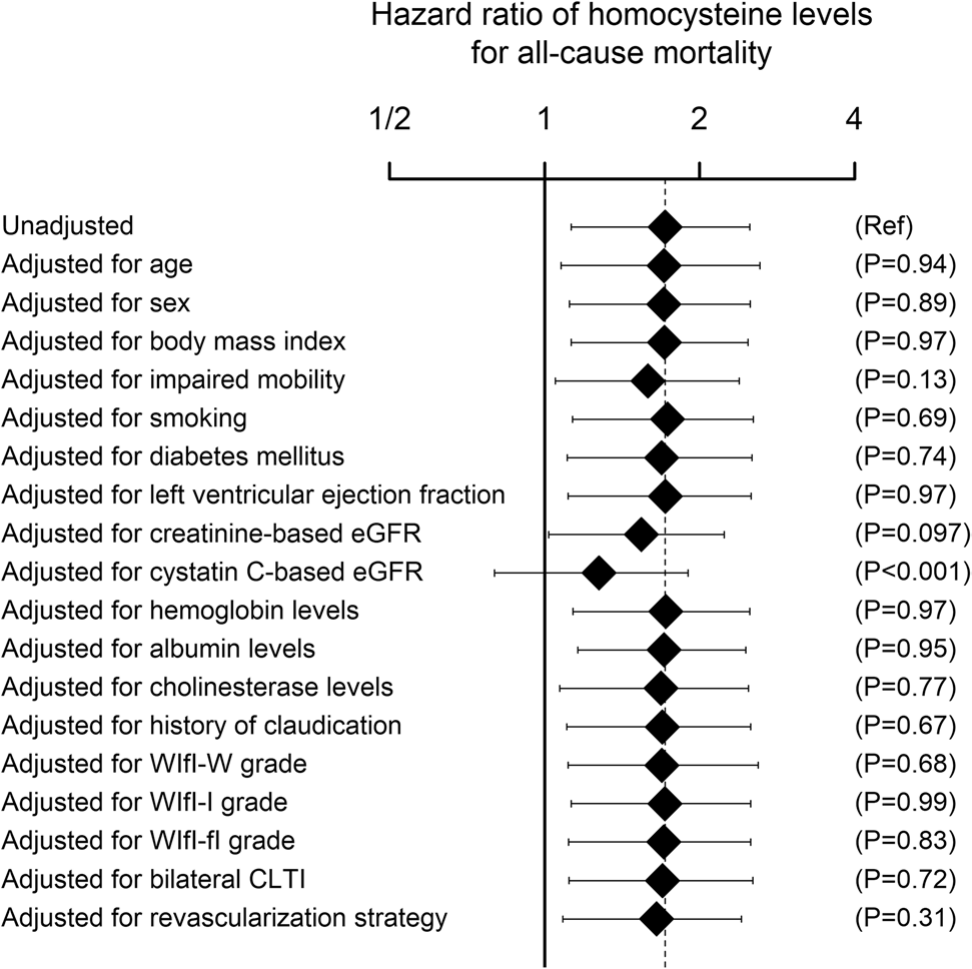
Hazard ratio of homocysteine levels for all-cause mortality. Data are hazard ratios of homocysteine levels (per twofold increase) for all-cause mortality, with adjustment for each covariate. Error bars indicate 95% confidence intervals. The dotted line as well as the top diamond shows the unadjusted hazard ratio of homocysteine levels (per twofold increase) for all-cause mortality. *P* values are for the difference between the hazard ratio adjusted for respective covariates and the unadjusted hazard ratio

During a median follow-up period of 2.8 (interquartile range 1.4–3.0) years, 62 patients died, and 65 patients experienced major adverse limb events, while 5 patients underwent major amputation. The univariate Cox proportional hazards regression model demonstrated that homocysteine levels were significantly associated with the mortality risk (*P* = 0.017); the unadjusted hazard ratio was 1.71 (1.13–2.50) per twofold increase. The hazard ratios adjusted for respective clinical characteristics are demonstrated in Fig. 1. The hazard ratio was significantly attenuated when adjusted for cystatin C-based eGFR (*P* < 0.001); the hazard ratio adjusted for cystatin C-based eGFR was 1.28 (0.80–1.90; *P* = 0.29). On the other hand, the hazard ratio adjusted for creatinine-based eGFR was 1.54 (1.02–2.23; *P* = 0.042), which was not significantly different from the unadjusted hazard ratio (*P* = 0.097). The hazard ratio was not significantly changed from the unadjusted one when adjusted for any of the other clinical characteristics (Fig. 1). The hazard ratio simultaneously adjusted for age, smoking, diabetes, and creatinine- and cystatin C-based eGFRs was 1.26 (0.74–2.02; *P* = 0.41). Homocysteine levels were not significantly associated with the risk of major amputation or that of major adverse limb events; the unadjusted hazard ratios per twofold increase were 1.50 (0.26–5.21; *P* = 0.56) and 0.97 (0.68–1.38; *P* = 0.86), respectively.

## Discussion

The current study demonstrated the clinical impact of plasma homocysteine levels in non-dialysis-dependent CLTI patients undergoing revascularization. Homocysteine levels were significantly associated with cystatin C-based eGFR. Homocysteine levels were significantly associated with an increased risk of mortality, but the association was attenuated when adjusted for cystatin C-based eGFR.

Homocysteine is an endogenous sulfur-containing amino acid intermediate of the essential amino acid methionine, and is not obtained directly from the diet [12]. An excessive amount of homocysteine will increase the oxidative stress and induce endothelial dysfunction and vessel injury, potentially accelerating atherosclerosis [13]. Its elevated plasma levels have been, therefore, expected to reflect systemic pro-atherosclerotic status and to be a useful biomarker related to cardiovascular disease [12]. On the other hand, in vivo, circulating homocysteine is filtered and metabolized by the kidneys in large amounts; decreased renal function will elevate plasma homocysteine levels [12]. The current finding of an inverse correlation between plasma homocysteine levels and eGFR was in line with this story.

Plasma homocysteine levels were associated with an increased mortality risk, but the association was attenuated after adjustment for cystatin C-based eGFR. This attenuation suggests that the prognostic impact of elevated plasma homocysteine levels would be explained by decreased renal function. Renal dysfunction is a well-known predictor of poor prognosis in a CLTI population [14], and hyperhomocysteinemia might be just a marker of renal dysfunction. In the prediction of the mortality risk, homocysteine levels might not provide more information than was provided by renal function. Future studies with a larger sample size will be needed to validate the current findings.

On the other hand, elevated plasma homocysteine levels were still significantly associated with the mortality risk when instead adjusted for creatinine-based eGFR. Creatinine-based eGFR would be inferior to cystatin C-based eGFR in accurate estimation of renal function [15]. Plasma homocysteine levels, as a marker of renal dysfunction, would compensate creatinine-based eGFR for its inaccuracy, and keep their significant association with the mortality risk in the bivariate model including creatinine-based eGFR as a covariate.

The current study had some limitations. First, data were not available on clinical variables potentially influencing plasma homocysteine levels, such as folate and B vitamin deficiencies, their supplementation, and the dietary consumption of methionine-containing protein. Second, the glomerular filtration rate was not directly measured, but was estimated with the use of creatinine and cystatin C levels. Third, the current study analyzed Japanese CLTI patients. Future studies on other ethnics will be needed to validate the current findings.

In conclusion, the current study investigated the clinical impact of plasma homocysteine levels in non-dialysis-dependent CLTI patients undergoing revascularization. The association of homocysteine levels with the mortality risk lost statistical significance after adjustment for cystatin C-based eGFR. An apparent association of homocysteine levels with an increased risk of mortality could be explained by renal dysfunction. Future studies will be needed to validate the current findings.
